# Early Onset Intrahepatic Cholangiocarcinoma: Clinical Characteristics, Oncological Outcomes, and Genomic/Transcriptomic Features

**DOI:** 10.1245/s10434-024-15013-5

**Published:** 2024-02-12

**Authors:** Diamantis I. Tsilimigras, Xu Han, Alfredo Guglielmi, Luca Aldrighetti, Matthew Weiss, Todd W. Bauer, Sorin Alexandrescu, George A. Poultsides, Shishir K. Maithel, Hugo P. Marques, Guillaume Martel, Carlo Pulitano, Feng Shen, François Chaucy, Bas Groot Koerkamp, Itaru Endo, Kazunari Sasaki, Federico Aucejo, Xu-Feng Zhang, Hua Zhu, Timothy M. Pawlik

**Affiliations:** 1 Department of Surgery, The Ohio State University Wexner Medical Center and James Comprehensive Cancer Center, Columbus, OH USA; 2 Department of Pancreatic Surgery, Zhongshan Hospital, Fudan University, Shanghai, China; 3 Department of Surgery, University of Verona, Verona, Italy; 4 Department of Surgery, Ospedale San Raffaele, Milan, Italy; 5 Department of Surgery, Johns Hopkins Hospital, Baltimore, MD USA; 6 Department of Surgery, University of Virginia, Charlottesville, VA USA; 7 Department of Surgery, Fundeni Clinical Institute, Bucharest, Romania; 8 Department of Surgery, Stanford University, Stanford, CA USA; 9 Department of Surgery, Emory University, Atlanta, GA USA; 10 Department of Surgery, Curry Cabral Hospital, Lisbon, Portugal; 11 Department of Surgery, University of Ottawa, Ottawa, Canada; 12 Department of Surgery, Royal Prince Alfred Hospital, University of Sydney, Sydney, Australia; 13 Department of Surgery, Eastern Hepatobiliary Surgery Hospital, Shanghai, China; 14 Department of Hepatobiliopancreatic Surgery and Liver Transplantation, AP-HP, Beaujon Hospital, Clichy, France; 15 Department of Surgery, Erasmus University Medical Centre, Rotterdam, The Netherlands; 16 Department of Gastroenterological Surgery, Yokohama City University School of Medicine, Yokohama, Japan; 17 Department of General Surgery, Digestive Disease and Surgery Institute, Cleveland Clinic, Cleveland, USA; 18 Department of Hepatobiliary Surgery, Institute of Advanced Surgical Technology and Engineering, The First Affiliated Hospital of Xi’an Jiaotong University, Xi’an, China

**Keywords:** Early onset, Prognosis, Intrahepatic cholangiocarcinoma, Resection, Genetic

## Abstract

**Introduction:**

Data on clinical characteristics and disease-specific prognosis among patients with early onset intrahepatic cholangiocarcinoma (ICC) are currently limited.

**Methods:**

Patients undergoing hepatectomy for ICC between 2000 and 2020 were identified by using a multi-institutional database. The association of early (≤50 years) versus typical onset (>50 years) ICC with recurrence-free (RFS) and disease-specific survival (DSS) was assessed in the multi-institutional database and validated in an external cohort. The genomic and transcriptomic profiles of early versus late onset ICC were analyzed by using the Total Cancer Genome Atlas (TCGA) and Memorial Sloan Kettering Cancer Center databases.

**Results:**

Among 971 patients undergoing resection for ICC, 22.7% (*n* = 220) had early-onset ICC. Patients with early-onset ICC had worse 5-year RFS (24.1% vs. 29.7%, *p* < 0.05) and DSS (36.5% vs. 48.9%, *p* = 0.03) compared with patients with typical onset ICC despite having earlier T-stage tumors and lower rates of microvascular invasion. In the validation cohort, patients with early-onset ICC had worse 5-year RFS (7.4% vs. 20.5%, *p* = 0.002) compared with individuals with typical onset ICC. Using the TCGA cohort, 652 and 266 genes were found to be upregulated (including ATP8A2) and downregulated (including UTY and KDM5D) in early versus typical onset ICC, respectively. Genes frequently implicated as oncogenic drivers, including CDKN2A, IDH1, BRAF, and FGFR2 were infrequently mutated in the early-onset ICC patients.

**Conclusions:**

Early-onset ICC has distinct clinical and genomic/transcriptomic features. Morphologic and clinicopathologic characteristics were unable to fully explain differences in outcomes among early versus typical onset ICC patients. The current study offers a preliminary landscape of the molecular features of early-onset ICC.

**Supplementary Information:**

The online version contains supplementary material available at 10.1245/s10434-024-15013-5.

The incidence of cancer in adolescents and young adults (AYAs) has dramatically increased over the past decade.^1^ While the reason for this increase is undoubtedly multifactorial, the increase in cancer among AYAs may be the result of delays in diagnosis because of higher uninsured rates, as well as delayed detection/suspicion given the traditional rarity of cancers in this age cohort.^2,3^ Adolescents and young adult patients with breast cancer are more commonly diagnosed at an advanced stage than older patients.^1^ Adolescents and young adults with cancer also may exhibit unique clinical characteristics and biologic behavior compared with individuals with typical onset disease.^4^ For example, AYAs with colorectal cancer are more likely to exhibit signet-ring histology and present at a more advanced stage, while also having lower rates of KRAS, NRAS, and BRAF mutations versus older individuals.^5,6^

Intrahepatic cholangiocarcinoma (ICC) is the second most common primary liver malignancy with an increasing incidence over the past three decades in the United States and worldwide.^7,8^ Although median age at the time of diagnosis is between 67 and 72 years, the incidence of ICC has increased annually among individuals younger than 50 years of age.^9,10^ Despite advances in defining ICC pathogenesis and natural history, age-specific differences in the biologic behavior of this tumor may exist.^11^ Feng et al. recently reported that AYAs with cholangiocarcinoma were more likely to carry additional sex combos, such as 1 (ASXL1) and lysine methyltransferase 2c (KMT2C) mutations compared with older patients with cholangiocarcinoma.^11^ As such, better characterization of potential differences in clinical presentation and genomic profiling of early- versus late-onset ICC are needed and may contribute to understanding differences in pathogenesis and prognosis.^8,12,13^

To date, there is a gap in knowledge relative to ICC incidence, clinical characteristics, and disease-specific prognosis of patients with early onset disease. Even less is known about potential genomic differences among patients with early (≤50 years) versus late/typical onset (>50 years) ICC. Therefore, the objective of the current study was to define the clinical characteristics, incidence, and outcomes of patients undergoing curative-intent resection of early- versus late-onset ICC. In addition, we sought to characterize unique genomic and transcriptomic features of early- versus late-onset ICC that may drive variations in prognosis after surgical resection.

## Methods

### Study Cohort, Inclusion/Exclusion Criteria

Patients who underwent curative-intent liver resection for ICC between 2000 and 2020 were identified by using the international, multi-institutional ICC study group database.^14,15^ Patients who underwent palliative resection, had R2 resection margins, had missing data on patient age, or had missing follow-up data were excluded (Supplemental Fig. 1). The institutional review board of all participating institutions approved this study.

### Clinicopathologic Variables and Outcomes

Variables of interest included age, sex, American Society of Anesthesiologists (ASA) class, preoperative serum CA19-9, etiology of ICC (i.e., hepatitis, stone, conventional ICC),^16^ presence of cirrhosis, tumor location (i.e., unilobar or bilobar), extent of resection (i.e., minor or major), AJCC 8^th^ edition T- and N-stage, tumor size, number of tumors (i.e., single, multiple), resection margin status (i.e., R0, R1), morphologic subtype (i.e., MF: mass-forming; IG: intraductal growth; or PI: periductal infiltrating), differentiation grade, presence of microvascular invasion or major vascular invasion, and receipt of adjuvant chemotherapy. Major hepatectomy was defined as resection of three or more Couinaud segments.^17^ Major vascular invasion was defined as invasion of the first- and second-order branches of the portal vein or hepatic arteries, or as invasion of one or more of the three hepatic veins. Microvascular invasion was defined as intraparenchymal vascular involvement identified on histological examination.^18^

The primary independent variable was age at the time of diagnosis, which categorized patients into early (age ≤50 years) and late onset ICC (age >50 years) groups.^19^ The primary outcomes were recurrence-free survival (RFS) and disease-specific survival (DSS). Recurrence-free survival was defined as the time interval between the date of hepatectomy and the date of recurrence or last follow-up. Disease-specific survival was defined as the time interval between the date of liver resection and the date of death from disease or last follow-up.

### External Validation Cohort

Data on patients who underwent curative-intent hepatectomy for ICC at two different institutions (Cleveland Clinic, Cleveland, OH, and First Affiliated Hospital of Xi'an Jiaotong University, Xi'an, China) were used to validate the association of early-/late-onset ICC with RFS and DSS. The external validation cohort included patients who met the same inclusion criteria as the patients in the test cohort.

### The Cancer Genome Atlas (TCGA) Cohort

To investigate the genomic and transcriptomic features of early versus late onset ICC patients, data on the genomic profile of ICC specimens were extracted from the TCGA, which is publicly available.^20^ Data from patients with ICC (ICD code C22.1) and available information on age and genomic/transcriptomic signatures were analyzed. TCGA data from HCC samples (ICD code C22.0) also were identified and were analyzed as a control group to ensure differences in early versus late onset ICC genomic signatures were unique and did not overlap with HCC samples.

### Genomic and Transcriptomic Analysis

RNA sequencing and single nucleotide polymorphism (SNP) mutation data were analyzed. The R package limma was applied to identify differentially expressed genes (DEGs) between early versus late onset ICC samples. Genes with a cutoff of |logFC| ≥ 1.0 and adj. *p* < 0.05 were defined as DEGs. The DEGs were visualized with the use of a volcano plot and a heatmap by using the ggplot2 package.^21^ The same analysis was performed on HCC samples. The overlapping DEGs between ICC and HCC samples were visualized with the use of a Venn diagram. Gene set enrichment analysis (GSEA) was performed to determine whether an *a priori* defined set of genes demonstrates statistically significant, concordant differences between two biological states. The functional and pathway enrichment knowledge of the DEGs were provided by Kyoto Encyclopedia of Genes and Genomes (KEGG, available online: http://www.kegg.jp/) database. In addition, somatic SNP mutation analysis was performed, and results were visualized by using the R maftools package.

### Exon Sequencing Data from Memorial Sloan Kettering Cancer Center

Exons sequencing data from the Memorial Sloan Kettering Cancer Center (MSKCC) ICC cohort with full bioinformation (*n* = 123; early onset: *n* = 16, late onset: *n* = 107) were available on cBioPortal (https://www.cbioportal.org/study/summary?id=ihch_mskcc_2020) and were further analyzed. Somatic genomic alterations, focusing on clinically relevant oncogenic drivers, such as single nucleotide variations and insertion-deletion mutations (indels), were analyzed to further assess differences between early and late onset ICC.

### Statistical Analysis

Continues and categorical variables were presented as median (interquartile range [IQR]) and frequency (%), respectively. Bivariable analyses included the Wilcoxon rank-sum test for continuous and chi-squared test or Fisher’s exact test for categorical variables, as appropriate. Differences in RFS and DSS between patients with early versus late onset ICC were compared by using the Kaplan-Meier method and the log-rank test in the multi-institutional dataset and later validated in an external validation cohort. Multivariable Cox regression analysis was performed to assess the impact of early versus late onset on RFS after adjusting for competing factors. The level of statistical significance for all tests was set at α = 0.05. All relevant statistical analyses were performed with the SPSS, v26 (IBM Corp. Armonk, NY) and JMP v16 (SAS Institute Inc., Cary, NC) statistical packages. The genomic and transcriptomic analysis was performed using R (R Project for Statistical Computing, Vienna, Austria), as described above.

## Results

### Clinical Presentation of Patients with Early Versus Late Onset ICC

A total of 971 patients met inclusion criteria and were included in the multi-institutional cohort (Table 1). Median patient age was 60 years (interquartile range [IQR] 51–69). Most patients were male (*n* = 537, 55.4%) and had an ASA class ≤ 2 (*n* = 685, 70.6%). Most patients had conventional ICC (*n* = 742, 76.4%), followed by hepatitis-related ICC (*n* = 163, 16.8%) and stone-related ICC (*n* = 66, 6.8%). The majority of patients had T1/T2 stage tumors (*n* = 804, 82.8%), whereas only 18.1% (*n* = 162) of patients had lymph node metastasis (N1). Most patients underwent an R0 resection (*n* = 840, 87.0%) and 30.9% (*n* = 289) of patients received adjuvant chemotherapy (Table 1).

**Table 1:** Baseline characteristics of patients with early- (≤50 years) vs. late-onset (>50 years) ICC

Variable	Overall (*n* = 971)	Early-onset ICC (*n* = 220, 22.7%)	Late-onset ICC (*n* = 751, 77.3%)	*p*
Age, median (IQR)	60 (51–69)	45 (41–48)	64 (57–71)	**<0.001**
Sex				0.08
Male	537 (55.4%)	133 (60.5%)	404 (53.9%)	
Female	433 (44.6%)	87 (39.5%)	346 (46.1%)	
ASA class > 2	241 (29.4%)	33 (16.2%)	208 (33.7%)	**<0.001**
CA19-9, UI/mL	47 (17–190)	39.5 (14.8–174.5)	53.0 (17.7–197.0)	0.12
Obesity*	119 (15.2%)	24 (12.2%)	95 (16.2%)	0.18
Etiology/ICC type				**<0.001**
Hepatitis ICC	163 (16.8%)	70 (31.8%)	93 (12.4%)	
Stone ICC	66 (6.8%)	10 (4.5%)	56 (7.5%)	
Conventional ICC	742 (76.4%)	140 (63.7%)	602 (80.1%)	
Cirrhosis	107 (12.1%)	41 (21.0%)	66 (9.5%)	**<0.001**
Location				0.21
Unilobar	734 (83.0%)	182 (85.8%)	552 (82.1%)	
Bilobar	150 (17.0%)	30 (14.2%)	120 (17.9%)	
Type of resection				**<0.001**
Minor resection	380 (43.1%)	123 (58.0%)	257 (38.4%)	
Major resection	502 (56.9%)	89 (42.0%)	413 (61.6%)	
AJCC 8th edition T stage				**0.006**
T1	441 (45.4%)	106 (48.2%)	335 (44.6%)	
T2	363 (37.4%)	89 (40.5%)	274 (36.5%)	
T3	89 (9.2%)	7 (3.2%)	82 (10.9%)	
T4	78 (8.0%)	18 (8.2%)	60 (8.0%)	
AJCC 8th edition N stage				**0.009**
N0	237 (26.5%)	39 (18.4%)	198 (29.0%)	
N1	162 (18.1%)	43 (20.3%)	119 (17.4%)	
Nx	495 (55.4%)	130 (61.3%)	365 (53.5%)	
Tumor size (cm)	6.0 (4.0–8.4)	6.3 (4.0–8.7)	6.0 (4.0–8.3)	0.49
Multiple tumors	155 (16.0%)	32 (14.5%)	123 (16.4%)	0.60
Margin status				0.10
R0	840 (87.0%)	198 (90.4%)	642 (86.1%)	
R1	125 (13.0%)	21 (9.6%)	104 (13.9%)	
Morphologic type				
MF, IG	821 (89.2%)	195 (91.5%)	626 (88.5%)	0.22
PI, MF+PI	99 (10.8%)	18 (8.5%)	81 (11.5%)	
Grade				
Well/moderate	747 (81.6%)	175 (83.3%)	572 (81.0%)	0.45
Poor/undifferentiated	169 (18.4%)	35 (16.7%)	134 (19.0%)	
Major vascular invasion	138 (14.3%)	31 (14.2%)	107 (14.3%)	0.95
Microvascular invasion	312 (32.6%)	51 (23.4%)	261 (35.3%)	**<0.001**
Adjuvant chemotherapy	289 (30.9%)	56 (26.0%)	233 (32.4%)	0.08

*IQR* interquartile range; *ASA* American Society of Anesthesiologist; *CA* carbohydrate antigen; *MF* mass-forming; *IG* intraductal growth; *PI* periductal infiltrating

*Among 783 patients with available data on BMI

Bold *p*-values denote statistical significance

Overall, 22.7% (*n* = 220) of patients had early-onset ICC, whereas 77.3% (*n* = 751) of patients had late-onset ICC. Etiology of ICC varied according to age at diagnosis; patients with early-onset ICC more frequently had hepatitis-related ICC (31.8% vs. 12.4%) and were less likely to have stone-related (4.5% vs. 7.5%) or conventional ICC (63.7% vs. 80.1%) versus individuals presenting with late onset ICC (*p* < 0.001). In addition, early-onset ICC patients more frequently had a history of cirrhosis (21.0% vs. 9.5%), T1/T2 tumors (88.7% vs. 81.1%), and N1 disease (20.3% vs. 17.4%), whereas late-onset ICC patients more frequently had undergone major resection (61.6% vs. 42.0%) and had microvascular invasion (35.3% vs. 23.4%) (all *p* < 0.05; Table 1). No differences were noted regarding preoperative CA19-9 levels, morphologic type, incidence of R0 resection margins, tumor grade, major vascular invasion, or utilization of adjuvant chemotherapy (all *p* > 0.05; Table 1). In addition, body mass index was comparable among patients with early- (median 24.5; IQR 22.0–27.4) versus late-onset ICC (median 25.2; IQR 22.3–28.0) (*p* = 0.08), as was the incidence of obesity between the two groups (early-onset ICC 12.2% vs. late-onset ICC 16.2%, *p* = 0.18).

### Multi-institutional Cohort: Impact of Early- Versus Late-Onset ICC on RFS and DSS

**Fig. 1:**
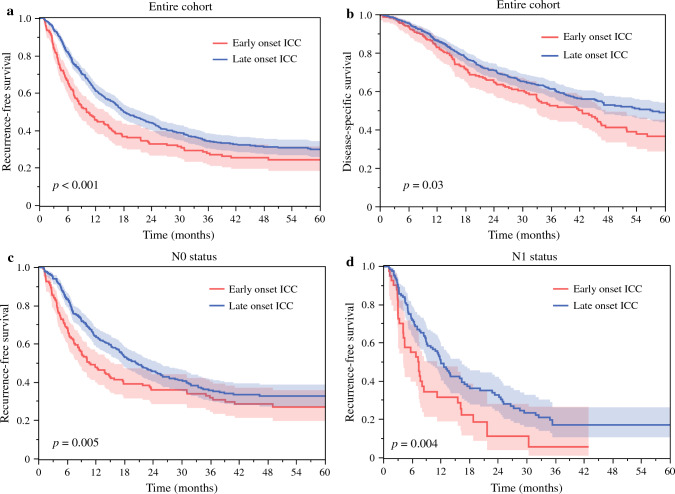
KM curves demonstrate differences in RFS (**a**) and DSS (**b**) between patients with early- versus late-onset ICC. KM curves demonstrate differences in RFS between patients with early- versus late-onset ICC stratified by N0 (**c**) and N1 (**d**) nodal status

After a median follow-up of 21.2 months (IQR 11.1–40.2), 5-year RFS and DSS following curative-intent liver resection for ICC were 28.5% and 45.5% among the entire cohort, respectively. Of note, patients with early-onset ICC had worse 6-month (66.7% vs. 81.9%), 2-year (32.6% vs. 43.9%), and 5-year RFS (24.1% vs. 29.7%) versus patients with late-onset ICC (*p* < 0.001; Fig. 1a). Similarly, individuals with early-onset ICC had worse 5-year DSS compared with individuals with late onset ICC (36.5% vs. 48.9%, *p* = 0.03; Fig. 1b). When stratified by nodal status, among patients with node-negative disease (N0), individuals with early-onset ICC had worse 2-year (36.0% vs. 45.7%) and 5-year RFS (27.0% vs. 32.8%) compared with individuals with typical-onset disease (*p* = 0.005; Fig. 1c). Similarly, among patients with metastatic nodal disease (N1), individuals with early-onset ICC had worse 2-year RFS (11.2% vs. 32.8%) compared with individuals with typical-onset ICC (*p* = 0.004; Fig. 1d). When stratified by geographic area, patients with early-onset ICC still had worse 2-year RFS compared with individuals with typical-onset ICC irrespective of whether they were treated at a Western (24.6% vs. 40.6%, *p* < 0.001) or Eastern institution (37.5% vs. 49.9%, *p* = 0.01).

On multivariable analysis, after adjusting for competing factors, patients with early-onset ICC had 49% higher hazards of recurrence after ICC resection (referent late-onset ICC; hazard ratio [HR] = 1.49, 95% confidence interval [CI] 1.17–1.89, *p* < 0.001) compared with individuals presenting with late-onset ICC (Table 2).

**Table 2:** Bivariable and multivariable analysis of recurrence-free survival (RFS)

Variables	Recurrence-free survival			
	Bivariate		Multivariable	
	HR, 95% CI	*p*	HR, 95%CI	*p*
Early-onset ICC	1.39 (1.16–1.67)	**<0.001**	1.49 (1.17–1.89)	**<0.001**
Sex (male)	1.07 (0.91–1.26)	0.43	–	–
ASA class (>2)	1.26 (1.04–1.52)	**0.02**	0.95 (0.70–1.31)	0.76
Obesity	1.39 (1.09–1.79)	**0.009**	2.14 (1.54–2.99)	**<0.001**
Cirrhosis	1.07 (0.82–1.40)	0.60	–	–
Etiology/ICC type				
Hep ICC	Ref		–	–
Stone ICC	1.11 (0.77–1.61)	0.57	–	–
Conventional ICC	1.03 (0.83–1.29)	0.79	–	–
CA 19-9 (>37), UI/mL	1.49 (1.23–1.80)	**<0.001**	1.32 (1.05–1.67)	**<0.001**
AJCC 8th edition T stage				
T1a/T1b	Ref		Ref	
T2/T3/T4	1.89 (1.58–2.21)	**<0.001**	1.69 (1.34–2.14)	**<0.001**
AJCC 8th edition N stage				
N0	Ref		Ref	
N1	1.68 (1.31–2.15)	**<0.001**	1.44 (1.04–1.99)	**0.03**
Nx	0.95 (0.78–1.16)	0.62	1.08 (0.78–1.49)	0.63
Margin status (R1)	1.25 (0.98–1.59)	0.07	–	–
Morphologic type				
MF, IG	Ref		Ref	
PI, MF+PI	1.30 (1.01–1.66)	**0.04**	0.91 (0.60–1.37)	0.64
Grade (poor/undiff)	1.55 (1.26–1.90)	**<0.001**	1.27 (0.93–1.72)	0.13
Major resection	1.18 (0.99–1.41)	0.06		
Adjuvant chemotherapy	1.50 (1.26–1.77)	**<0.001**	1.09 (0.81–1.48)	0.56

*ASA* American Society of Anesthesiologist; *CA* carbohydrate antigen; *MF* mass-forming; *IG* intraductal growth; *PI* periductal infiltrating; *AJCC* American Joint Committee on Cancer; *CA* carbohydrate antigen; *HR* hazard ratio; *CI* confidence interval

Bold *p*-values denote statistical significance

### External Validation Cohort: Early- Versus Late-Onset ICC

**Fig. 2:**
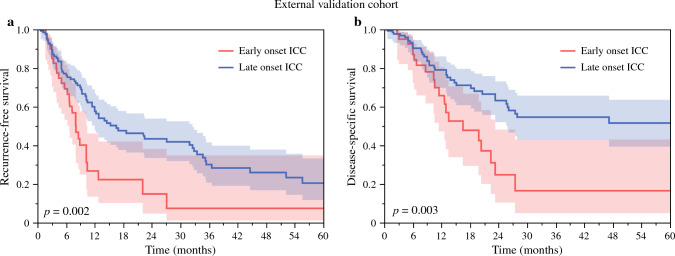
KM curves demonstrate differences in RFS (**a**) and DSS (**b**) between patients with early- versus late-onset ICC in the external validation cohort

The differential prognosis of patients undergoing curative-intent resection for early- versus late-onset ICC was validated by using an external cohort from the First Affiliated Hospital of Xi'an Jiaotong University (Xi'an, China) (*n* = 104) and Cleveland Clinic (Cleveland, OH) (*n* = 74). In the external validation cohort, 40 (22.5%) of patients had early-onset ICC, whereas 138 (77.5%) of patients had late-onset ICC. Survival analyses confirmed the results of the multi-institutional cohort. Specifically, patients with early-onset ICC had worse 5-year RFS versus individuals with late-onset ICC after curative-intent resection (7.4% vs. 20.5%, *p* = 0.002; Fig. 2a). Similarly, 5-year DSS was worse among patients with early- versus late-onset ICC after resection (16.5% vs. 51.6%, *p* = 0.003; Fig. 2b).

### Identification of DEGs Between Early- Versus Late-Onset ICC

**Fig. 3:**
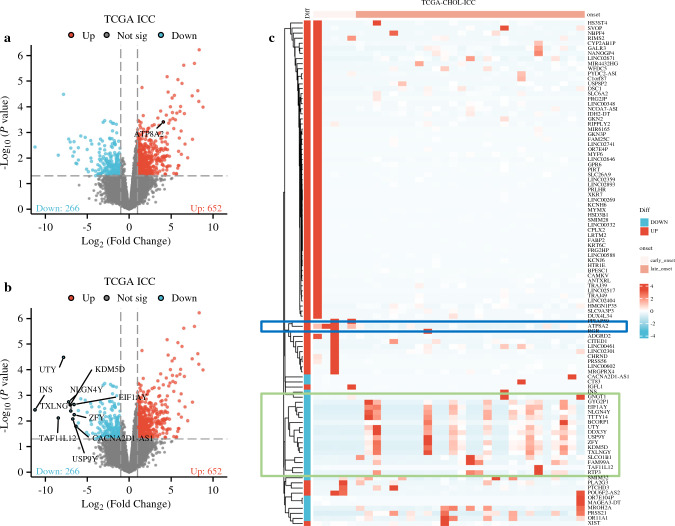
DEGs among early- versus late-onset ICC samples using data from the TCGA. Volcano plot demonstrates upregulation of 652 genes and downregulation of 266 genes between early-onset and late-onset ICC samples (noted upregulation of ATP8A2 and downregulation of top 10 genes) (**a, b**). Heatmap shows the top100 DEGs between early-onset and late-onset ICCs (**c**)

To elucidate possible genomic/transcriptomic variations that may be driving the differential prognosis of patients with early- versus late-onset ICC, the TCGA database was analyzed. The screened TCGA cohort with complete bioinformation consisted of 32 patients with ICC and known age (early-onset ICC: *n* = 5, 15.6% vs. late-onset ICC: *n* = 27, 84.4%) and 19 patients with unknown age. In analyzing DEGs among patients with known age and early- versus late-onset ICC using the TCGA cohort, 652 and 266 genes were noted to be upregulated and downregulated, respectively (Fig. 3a, b). The top 100 upregulated (including ATPase Phospholipid Transporting 8A2 [ATP8A2]) and downregulated genes (including UTY, KDM5D, INS, NLGN4Y, TXLNG, EIF1AY, ZFY, TAF11L12, CACNA2D1-AS1, USP9Y) for each ICC sample were identified (Fig. 3c). TCGA data from HCC samples (*n* = 370) also were classified in the TCGA and analyzed as control group (early-onset HCC: *n* = 78, 21.1% vs. late-onset HCC: *n* = 292, 78.9%). Similar analysis demonstrated 296 and 164 genes that were upregulated and downregulated in the early- versus late-onset HCC samples, respectively (Supplemental Fig. 2a). Of note, there was minimal overlap between DEGs that were up- or down-regulated among early- versus late-onset ICC and HCC samples (Supplemental Fig. 2b).

### Functional Enrichment Analysis

GSEA using KEGG database was then performed to examine possible functional roles of the identified DEGs. In KEGG enrichment analysis, DEGs were closely associated with citrate cycle (TCA cycle) and oxidative phosphorylation, carbon metabolism in cancer, and reactive oxygen species (ROS) pathways (Supplemental Fig. 3). Significant enrichment also was observed for nonalcoholic fatty liver disease and autophagy pathways.

### SNP Mutational Analysis of Top Ten Genes

**Fig. 4:**
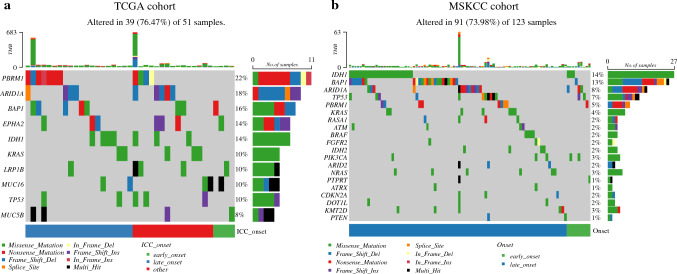
**a** The somatic single nucleotide polymorphism mutation analysis from the TCGA cohort among early-onset, late-onset, and other (i.e., unknown age) ICC samples. Waterfall plot shows the mutational frequency of the top ten genes and the respective mutation type for each ICC sample. **b** Exon sequencing mutation analysis from the MSKCC cohort reveals that genes frequently implicated as oncogenic drivers (i.e., BRAF, KRAS, FGFR2, CKDN2A, IDH1, IDH2) were infrequently mutated in early-onset ICC patients compared with late-onset ICC patients

Differences in SNP mutations of the top ten genes between early- and late-onset ICC were further examined. Interestingly, PBRM1 and ARID1A genes were mutated in 22% and 18% of the samples, respectively; of note, none of these SNP mutations were identified in early-onset ICC samples. The mutational frequency of KRAS and TP53 was 10% for each of these genes and, similarly, none was mutated in early-onset ICC samples (Fig. 4a).

### Exon Sequencing Data from the MSKCC Cohort

Exon sequencing data from MSKCC demonstrated that genes frequently implicated as oncogenic drivers, such as KRAS, BRAF, FGFR2 TP53, CDKN2A, IDH1, IDH2, and NRAS, were infrequently mutated in the early-onset ICC patients, whereas genes, such as BAP1, ARID1A, and PBRM1, that were mostly mutated in late-onset ICC, were rarely mutated in the early-onset ICC cohort (Fig. 4b). These data collectively suggest that early-onset ICC has a unique genomic and transcriptomic profile compared with late-onset ICC.

## Discussion

ICC is a rare malignancy arising from the intrahepatic bile ducts that has been generally associated with poor long-term outcomes.^8,12,13,22^ Median OS among patients with resectable tumors ranges from 15 to 40 months, whereas OS among individuals with unresectable ICC is only 6 to 13 months.^8,12,13^ Data on age-related differences in prognosis among patients with ICC are, however, currently lacking. In particular, whether early-onset ICC is clinically or biologically different from late-onset ICC remains not defined. The current study was important, because we specifically examined prognosis, as well as possible genetic differences, among patients who had early- versus late-onset ICC. Of note, using an international, multi-institutional cohort, we demonstrated that patients with early onset ICC had worse 5-year RFS and DSS versus individuals with late onset ICC following curative-intent resection; this finding was further validated in an external validation cohort. Importantly, early-onset ICC was associated with worse RFS even after adjusting for other clinicopathologic characteristics. In addition, early-onset ICC was associated with certain DEGs (including upregulation of ATP8A2 and downregulation of UTY, KDM5D, INS, etc.) compared with late-onset ICC samples. There also was minimal overlap between ICC and HCC tissue samples, suggesting a unique RNA sequencing expression profile for early-onset ICC. To our knowledge, this is the first study to characterize the outcomes, as well as the genomic features associated with early versus late-onset ICC.

Previous research has focused on identifying predictors of outcomes among individuals with ICC,^15,23^ yet there has been little emphasis on the age at diagnosis as a risk factor for adverse outcomes. A previous study from our group demonstrated that age at diagnosis was inversely associated with very early recurrence (i.e., within 6 months) following curative-intent resection for ICC.^23^ In specific, an 1-year increase in age at diagnosis was associated with 3% lower odds of developing very early recurrence after ICC resection after adjusting for competing factors (odds ratio [OR] 0.97, 95% CI 0.96–0.99).^23^ In line with these findings, the current study demonstrated that individuals with early-onset ICC (≤50 years of age) had worse 6-month (66.7% vs. 81.9%), 2-year (32.6% vs. 43.9%), and 5-year RFS (24.1% vs. 29.7%) versus patients undergoing resection for late-onset ICC (Fig. 1a, p < 0.001). These findings were consistent among patients with both N0 and N1 disease (Fig. 1c, d). This difference in prognosis was further validated in an external cohort in which differences in 5-year RFS among patients undergoing resection for early- versus late-onset ICC were even more pronounced (7.4% vs. 20.5%, *p* = 0.002; Fig. 2). Of note, while patients with early-onset ICC more frequently had a history of hepatitis (31.8% vs. 12.4%) and cirrhosis (21.0% vs. 9.5%), no differences in other clinicopathologic characteristics (i.e., resection margin status, morphologic type, tumor grade, major vascular invasion, or adjuvant chemotherapy utilization) were noted between the two groups that could potentially explain the stark difference in outcomes. Perhaps more interesting, early-onset ICC was associated with earlier T-stage tumors (T1/T2 tumors; 88.7% vs. 81.1%) and a lower incidence of microvascular invasion (23.4% vs. 35.3%) versus late-onset ICC, a finding that would generally suggest better anticipated long-term outcomes. Despite this, early-onset ICC was independently associated with 50% higher hazards of recurrence (HR 1.49, 95% CI 1.17–1.89) compared with late-onset ICC after curative-intent resection. Taken together, the current study strongly suggests that early onset ICC was associated with worse long-term outcomes versus the late-/typical-onset ICC. In addition, the data highlight how standard morphologic and clinicopathologic criteria were not successful in explaining the differences in long-term outcomes among patients with early- versus late-onset ICC. In turn, rather than morphology, disease biology is more likely the main driver of prognosis.

AYAs with cholangiocarcinoma might exhibit unique clinical characteristics and biologic behavior compared with individuals with late-/typical-onset disease; yet this area has been largely understudied.^4^ Recently, Feng et al. reported that AYAs with cholangiocarcinoma presented with a higher incidence of poorly differentiated disease and more advanced-stage tumors compared with older adults.^11^ In addition, AYAs with cholangiocarcinoma were more likely to carry ASXL1 and KMT2C mutations compared with older individuals.^11^ However, this previous study analyzed both intra- and extrahepatic cholangiocarcinoma tumors together. To date, no study has focused on the age-related genomic differences specific to ICC. The current study further defined the genomic and transcriptomic features of early versus late onset ICC and identified potential differences at the molecular level that could be contributing to prognostic differences. In particular, by utilizing the TCGA cohort, DEGs between early- and late-onset ICC samples were identified. Of note, 652 and 266 genes were noted to be up- and down-regulated, respectively. To validate that these findings were unique to ICC, an analysis of HCC samples was performed that yielded minimal to low overlap of DEGs between ICC and HCC samples. As such, the data suggested that early-onset ICCs have a unique RNA sequencing expression profile that is distinct from HCCs. Among the altered genes identified, ATP8A2 was upregulated in early-onset ICC samples (Fig. 3). The protein encoded by the ATP8A2 gene is a member of the P4 ATPase family of proteins and a component of the P4-ATPase flippase complex. This complex catalyzes the hydrolysis of ATP involved in the transport of aminophospholipids from the outer to the inner leaflets of diverse membranes and ensures that phospholipids maintain asymmetrical distribution.^24^ ATP8A2 is abnormally methylated in various cancer tissues and may contribute to cancer progression.^25^ Ding et al. reported an upregulation of circ-ATP8A2 in human cervical cancer tissue samples, which was associated with cancer cell progression via regulating miR-433/EGFR signaling pathway.^26^ Similarly, the ATP8A2 gene may play a role in facilitating more aggressive biologic behavior among patients with early-onset ICC. Based on the functional enrichment analysis, DEGs in early- versus late-onset ICC samples also were closely associated with oxidative phosphorylation and ROS pathways, which act as regulators of important signaling pathways in carcinogenesis and cancer progression.^27,28^ Of note, the SNP mutational analysis demonstrated that KRAS was mutated in 10% of ICC samples, of which none was early-onset ICC. These data are consistent with the previous literature, suggesting a low frequency of KRAS mutations in ICC tumors.^29,30^ In addition, exon sequencing data from the MSKCC cohort demonstrated that frequent known oncogenic drivers in ICC, such as IDH1, IDH2, CDKN2A, TP53, BRAF, FGFR2, and KRAS, were infrequently mutated in early-onset ICC versus late-onset ICC patients. Collectively, the data demonstrated a unique molecular signature of early-onset ICC compared with late-onset ICC and highlight potential mechanisms to explain a more aggressive tumor biology.

The results of the current study should be interpreted in light of certain limitations. Because of the retrospective nature of the study, selection bias was possible. Nevertheless, worse disease-specific outcomes among patients with early-onset ICC were noted both in the multi-institutional database, as well as in an external validation cohort. Similar results were noted when performing a subgroup analysis by Eastern versus Western experience in the multi-institutional cohort further supporting the generalizability of our findings. In addition, the clinical databases used to examine long-term outcomes relative to age at diagnosis included surgical patients only. Therefore, the results of the study may not necessarily be extrapolated to non-surgical populations. In addition, because of the limitations of the TCGA cohort, we were unable to assess the association between the identified DEGs with disease-specific outcomes (i.e., RFS, DSS) among patients with early onset ICC.

## Conclusions

Patients with early-onset ICC had distinct clinical characteristics and worse prognosis compared with individuals with late-onset ICC. Morphologic and clinicopathologic characteristics were unable to explain differences in outcomes among early- versus late-onset ICC patients. Early-onset ICC exhibited a unique genomic and transcriptomic profile distinct from late-onset ICC and HCC. Future, larger-scale studies are required to validate the results of the current study, as well as further define the molecular landscape of early-onset ICC.

### Supplementary Information

Below is the link to the electronic supplementary material.

Supplemental Fig. 1 Final study sample size after applying exclusion criteria (PDF 9 KB)

Supplemental Fig. 2 DEGs among early- versus late-onset HCC samples using data from the TCGA. Volcano plot demonstrates upregulation of 296 genes and downregulation of 164 genes between early-onset and late-onset HCC samples (a). Venn diagram shows minimal overlap of DEGs between early- and late-onset ICC and HCC samples (b) (PDF 249 KB)

Supplemental Fig. 3 Gene Set Enrichment Analysis (GSEA) using DEGs between early- versus late-onset ICC. Each dot represents one term. The dot size and color indicate the number of genes involved and the statistical significance. The dot plot shows the top 20 enrichment pathways between early-onset and late-onset ICCs (PDF 121 KB)
