# Supplementary material for: Early Onset Intrahepatic Cholangiocarcinoma: Clinical Characteristics, Oncological Outcomes, and Genomic/Transcriptomic Features
Source: Ann Surg Oncol. 2024 Feb 12;31(5):3087–97. doi: 10.1245/s10434-024-15013-5 (PMC10997729; doi:10.1245/s10434-024-15013-5)
Supplement: Supplementary file 1 — Supplemental Fig. 1 Final study sample size after applying exclusion criteria (PDF 9 KB) [file 10434_2024_15013_MOESM1_ESM.pdf]

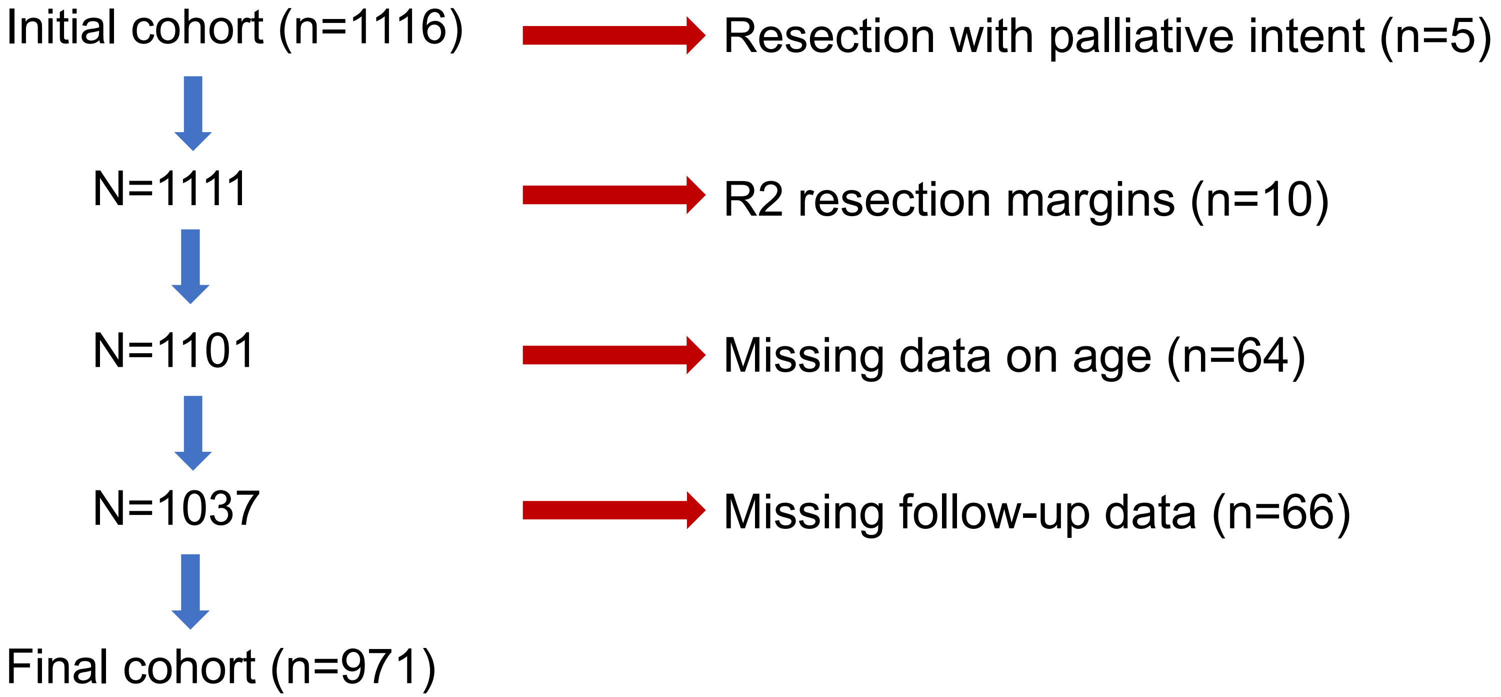
Initial cohort (n=1116)
Resection with palliative intent (n=5)
N=1111
R2 resection margins (n=10)
N=1101
Missing data on age (n=64)
N=1037
Missing follow-up data (n=66)
Final cohort (n=971)
