# Supplementary figures and images for: Early Onset Intrahepatic Cholangiocarcinoma: Clinical Characteristics, Oncological Outcomes, and Genomic/Transcriptomic Features
Source: Ann Surg Oncol. 2024 Feb 12;31(5):3087–97. doi: 10.1245/s10434-024-15013-5 (PMC10997729; doi:10.1245/s10434-024-15013-5)

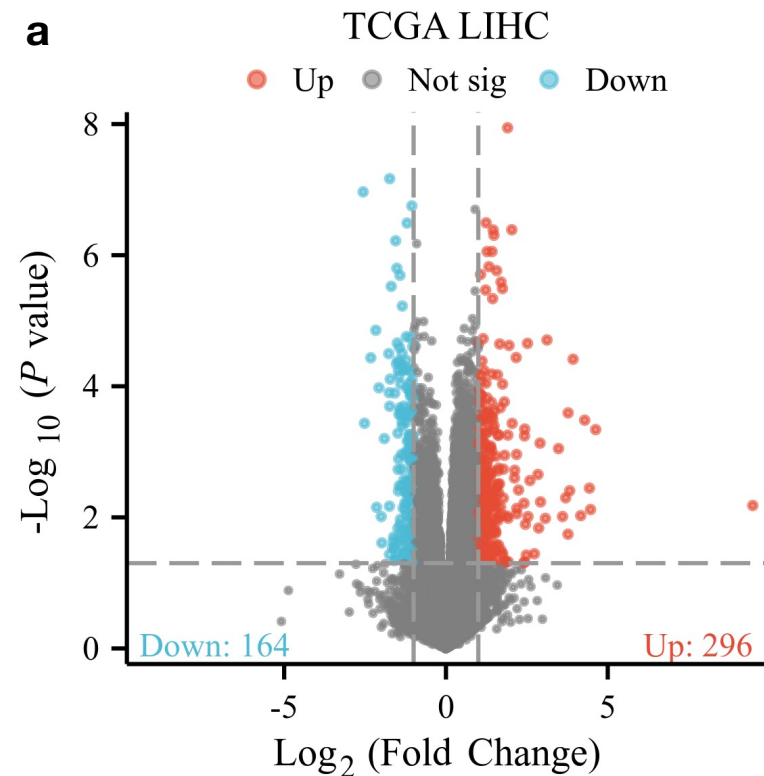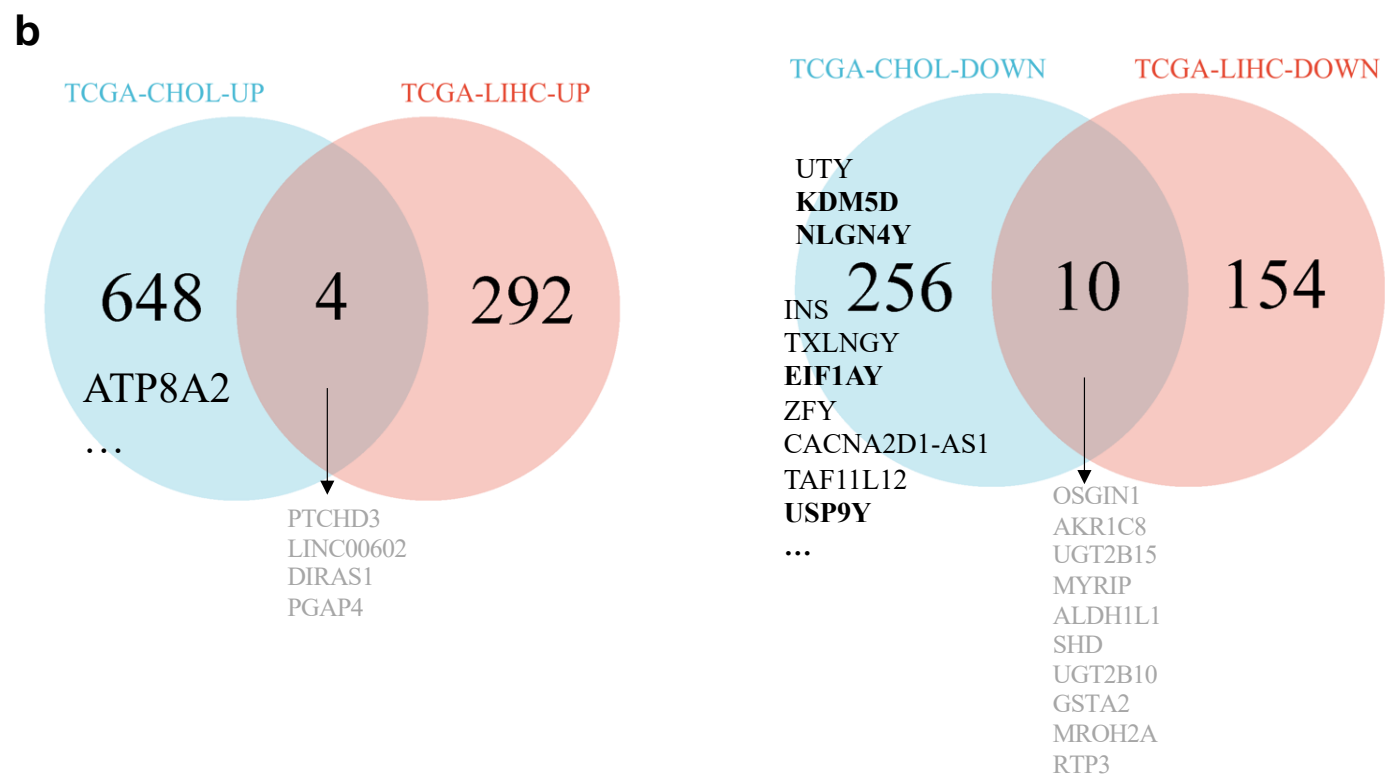

Supplement: Supplementary file 2 — Supplemental Fig. 2 DEGs among early- versus late-onset HCC samples using data from the TCGA. Volcano plot demonstrates upregulation of 296 genes and downregulation of 164 genes between early-onset and late-onset HCC samples (a). Venn diagram shows minimal overlap of DEGs between early- and late-onset ICC and HCC samples (b) (PDF 249 KB) [file 10434_2024_15013_MOESM2_ESM.pdf]

# KEGG early vs. late onset ICC

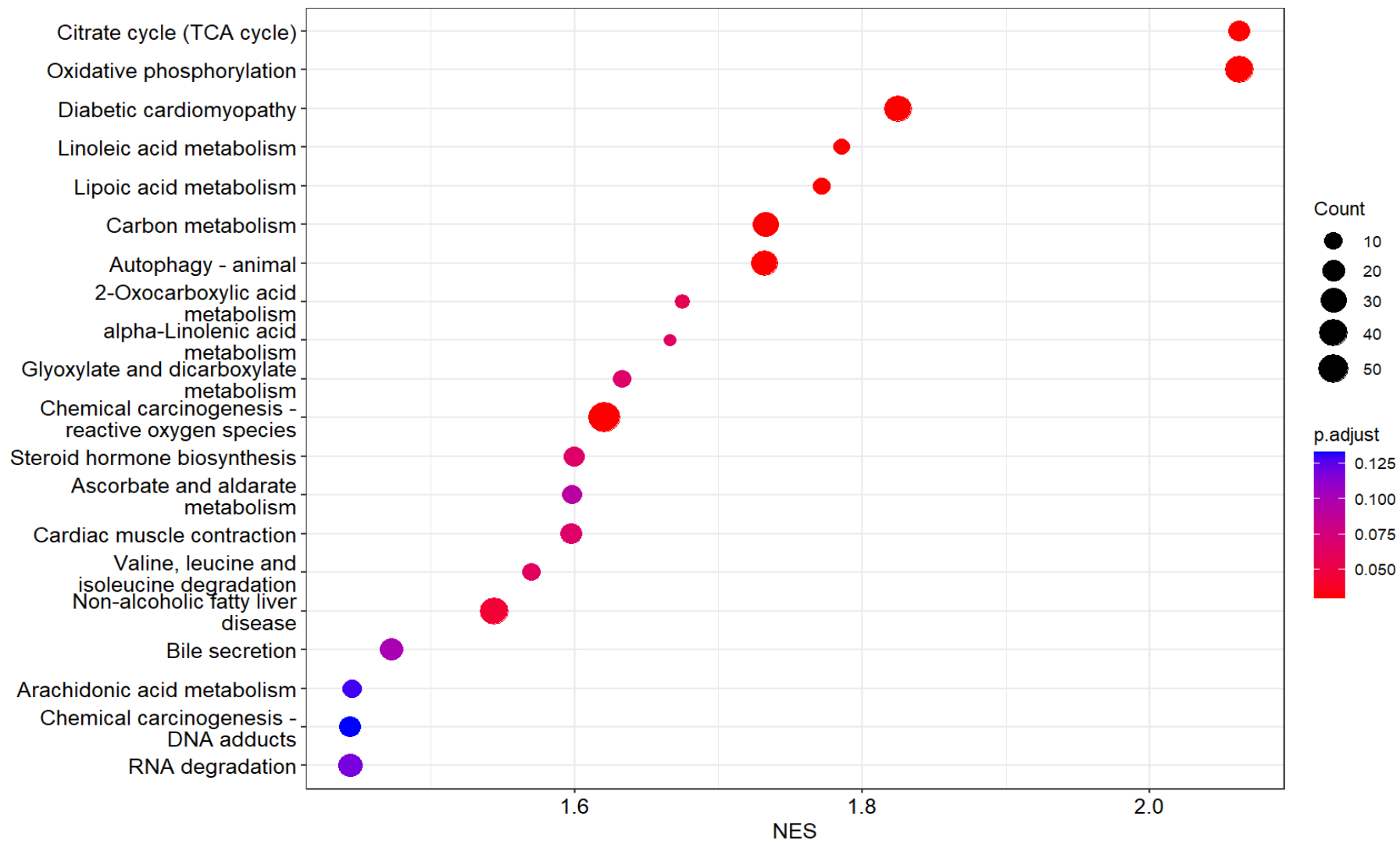

Supplement: Supplementary file 3 — Supplemental Fig. 3 Gene Set Enrichment Analysis (GSEA) using DEGs between early- versus late-onset ICC. Each dot represents one term. The dot size and color indicate the number of genes involved and the statistical significance. The dot plot shows the top 20 enrichment pathways between early-onset and late-onset ICCs (PDF 121 KB) [file 10434_2024_15013_MOESM3_ESM.pdf]
